# Management of Patients with Adhesive Capsulitis via Ultrasound-Guided Hydrodilatation without Concomitant Intra-Articular Lidocaine Infusion: A Single-Center Experience

**DOI:** 10.3390/life12091293

**Published:** 2022-08-23

**Authors:** Yung-Chieh Chen, Shu-Huei Shen, Hong-Jen Chiou, Yung-Liang Wan

**Affiliations:** 1 Department of Medical Imaging, Taipei Medical University Hospital, Taipei 11031, Taiwan; 2 Department of Radiology, Taipei Veterans General Hospital, National Yang Ming Chiao Tung University, Taipei 11217, Taiwan; 3 Department of Medical Imaging and Intervention, Linkou Chang Gung Memorial, College of Medicine, Chang Gung University, Taoyuan City 333423, Taiwan

**Keywords:** adhesive capsulitis, chondrotoxicity, intra-articular lidocaine, ultrasound-guided hydrodilatation

## Abstract

Considering the potential chondrotoxic effects of lidocaine, this retrospective study aimed to examine whether ultrasound-guided hydrodilatation without concurrent lidocaine infusion can still provide comparable treatment benefits for patients with adhesive capsulitis (AC). Outpatient data from 104 eligible AC patients who received ultrasound-guided hydrodilatation between May 2016 and April 2021 were reviewed. A total of 59 patients received hydrodilatation with diluted corticosteroid only, while 45 patients received treatment with mixed, diluted corticosteroid and 1% lidocaine. The overall treatment outcome was documented as the percentage of clinical improvement, ranging from 0% to 100% compared to baseline, and it was ranked into poor, moderate and good treatment outcomes. The results show no significant group-wise difference in demographics, overall treatment outcome, and number of hydrodilatations, while most patients showed moderate and good treatment outcomes. Patients with lidocaine infusion did not show greater treatment benefit. Our results suggest that ultrasound-guided hydrodilatation without concurrent lidocaine infusion can still deliver good treatment benefits for AC patients, and the findings are supportive of a modified approach toward careful intra-articular local anesthetic use during management of AC in the primary care setting.

## 1. Introduction

Adhesive capsulitis (AC), otherwise known as “frozen shoulder”, is a frequently described shoulder disorder among middle-aged adults manifested by painful, progressive limitation of shoulder motion [1]. The pathogenesis of AC involves idiopathic or secondary insults (such as glenohumeral surgery or trauma) that induce inflammation and proliferative fibrosis of the glenohumeral joint, ultimately leading to glenohumeral capsular contraction and reduced capsular volume [2,3]. Ultrasound-guided hydrodilatation is a well-established nonsurgical treatment option for the management of AC, and it can deliver excellent short-term functional outcomes in terms of joint mobilization and pain reduction [4]. The treatment is typically performed by injecting mixed, diluted corticosteroid and local anesthetic into the contracted glenohumeral joint space under ultrasound guidance to achieve hydraulic capsular distension (or rupture), as well as to reduce inflammation and pain, and it is commonly followed by physiotherapy to promote joint stretching and mobilization.

Recent in vitro and animal studies revealed growing concerns over the chondrotoxic properties of local anesthetics, including lidocaine hydrochloride [5,6,7]. In particular, an in vitro study indicated that a single dose of 1% lidocaine can significantly affect chondrocyte viability [8]. In consideration of these findings, it is clinically sensible to preserve the use of intra-articular lidocaine for patients with a greater need for pain control, thereby obviating the potential risk of iatrogenic cartilage injury in other patients whose symptom of joint pain could be managed by alternative therapeutic methods, such as physical therapy with oral analgesics. Nonetheless, this modified approach toward careful intra-articular local anesthetic use during hydrodilatation has not been examined previously. On the other hand, despite the widespread application of lidocaine during hydrodilatation throughout the literature, its necessity and significance to the procedure itself have not been well established [9], and previous studies using an increased dosage of intra-articular lidocaine did not demonstrate additional treatment benefit [10]. Therefore, the aim of this study was to assess and compare the general treatment outcome of ultrasound-guided hydrodilatation with or without concurrent intra-articular lidocaine infusion in patients with AC. We hypothesize that, with careful patient selection and clinical judgment, ultrasound-guided hydrodilatation without concomitant intra-articular lidocaine infusion can still deliver excellent treatment benefits for patients with AC, while obviating the potential risk of iatrogenic cartilage injury.

## 2. Materials and Methods

### 2.1. Patient Selection

This study was approved by the local institutional review board (TPEVGH IRB No.: 2021-11-017CC). We retrospectively reviewed the outpatient medical charts of patients with AC who received ultrasound-guided hydrodilatation at the radiologic outpatient clinic of Taipei Veterans General Hospital, a tertiary medical center in Taiwan, between May 2016 and April 2021 with the following inclusion criteria: (1) clinical and imaging diagnosis of AC with compatible clinical history and physical findings, including chronic shoulder pain level greater than 4/10 on the visual analog scale (VAS), and limitation of glenohumeral joint movement for over 3 months; (2) no significant improvement after conservative treatment; and (3) ability to participate in proactive shoulder motion exercises after hydrodilatation. Patients were excluded if (1) their chronic shoulder symptoms were attributed to other medical conditions such as cervical radiculopathy and tendinosis; (2) they were diagnosed with severe AC that requires surgical management; (3) they had concomitant shoulder diseases including joint infection, rotator cuff tear, and acute trauma; and (4) they had other contraindications for hydrodilatation treatment such as coagulopathy, systemic diseases, and an allergy toward corticosteroid or lidocaine. Patients were also excluded if no medical information regarding treatment outcome was available, or if they received intra-articular injection with other injectates such as sodium hyaluronate.

### 2.2. Treatment Process

**Figure 1:**
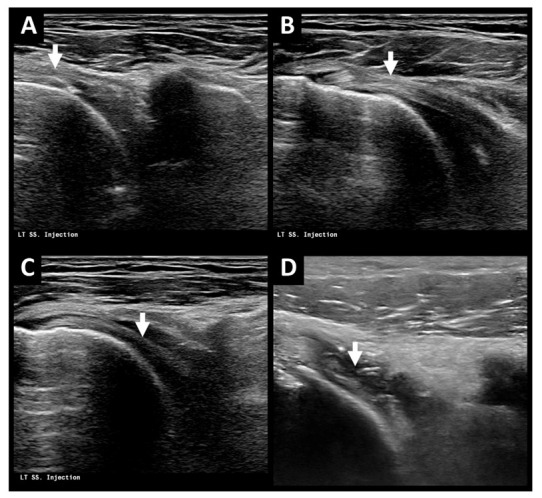
(**A**–**C**) Ultrasound **i**mages during ultrasound-guided hydrodilatation in a 69-year-old woman with AC of left shoulder via anterior rotator cuff interval approach with lateral in-plane view. Note visualization of the injection needle (white arrow, **A**), coracohumeral ligament (white arrow, **B**), and biceps tendon (white arrow, **C**) during needle placement. (**D**) Ultrasound **i**mage after ultrasound-guided hydrodilatation in a 54-year-old man with AC of left shoulder. Note the irregular and thickened glenohumeral joint capsule (white arrow) suggestive of capsulitis. AC = adhesive capsulitis.

The ultrasound-guided hydrodilatation procedure was performed by a senior radiologist (H.J. Chou, with 36 years of experience in ultrasound-guided injection treatment) using an Aplio i800 ultrasound system (Canon Medical Systems, Tochigi, Japan) with a 6–18 MHz linear ultrasound transducer via the anterior rotator cuff interval approach. During the treatment procedure, the patients were placed in a supine position with their target shoulder arm in extension–abduction. Upon visualization of the rotator cuff interval with ultrasound at the deltopectoral groove, a 23-gauge long injection needle was used under an aseptic technique to approach the glenohumeral joint space beneath the deep fascia of supraspinatus muscle via a lateral in-plane approach (Figure 1). Following successful needle positioning, intra-articular injection was performed slowly with an injectate consisting of 1 mL of 10 mg (10 mg/mL) triamcinolone acetonide (Shincort) diluted in 20 mL of normal saline, with or without additional 1% lidocaine hydrochloride (Xylocaine). The goal of injection was to gradually distend the glenohumeral joint capsule while avoiding capsular rupture. The use of intra-articular lidocaine was determined according to clinical judgment, symptom severity, and patient compliance, supported by shared decision making with the patient.

Lidocaine was not provided in patients who presented with relatively milder symptoms, expressed greater tolerance toward pain, or had known adverse reactions to lidocaine, as well as in patients who were concerned with the potential chondrotoxic effect of lidocaine during pretreatment discussions. On the other hand, lidocaine was provided for patients who presented with more severe symptoms (e.g., shoulder pain level greater than 7/10 on the VAS), were less tolerant towards pain, or demanded on-site pain relief regardless of symptom severity. For this group of patients, between 1 and 5 mL of 1% lidocaine was provided according to clinical demand and operator experience.

Immediately after hydrodilatation, both groups of patients underwent supervised shoulder motion exercises, including wall climbing, towel stretching, and pendulum exercises, and they were instructed to repeat these exercises at least twice a day at home for a minimum duration of 4 weeks. Oral analgesics including acetaminophen and nonsteroidal anti-inflammatory drugs (NSAIDs) were prescribed for pain control. Outpatient follow-up visits were arranged on a monthly basis, during which patients were re-evaluated for improvements in clinical symptoms such as shoulder pain and quality of life, as well as improvements in shoulder motion during physical examination. The assessment results were documented as the percentage of clinical improvement ranging from 0% (no improvement) to 100% (full recovery) compared to the pretreatment baseline. Repeated hydrodilatation using the same injectate regimen was performed according to clinical need and patient compliance. The overall treatment outcomes of the patients were determined by the percentage of clinical improvement obtained during their last outpatient visit or telemedicine appointment (corresponding to short-term post-hydrodilatation follow-up within 1–3 months for the majority of patients), and they were ranked into three categories: poor outcome (improvement < 50%), moderate outcome (improvement ≥ 50% and <80%), and good outcome (improvement ≥ 80%).

### 2.3. Statistical Analysis

The statistical analysis was performed with SPSS version 26.0 (SPSS Inc., Chicago, IL, USA). The differences in patient demographics were examined using the chi-square test for categorical variables, including gender and laterality of affected shoulder, and using the Student’s *t*-test for differences in age, with homogeneity of variance assessed via Levene’s test. The Mann–Whitney U test was used to examine group-wise differences in overall treatment outcome and number of hydrodilatation. Additionally, Spearman’s rank correlation was used to assess the association between the number of hydrodilatations and overall treatment outcome across all patients, as well as the correlation between the provided dose of intra-articular lidocaine and overall treatment outcome in the group of given patients. A *p*-value of <0.05 was considered statistically significant.

## 3. Results

### 3.1. Patient Selection and Demographics

**Figure 2:**
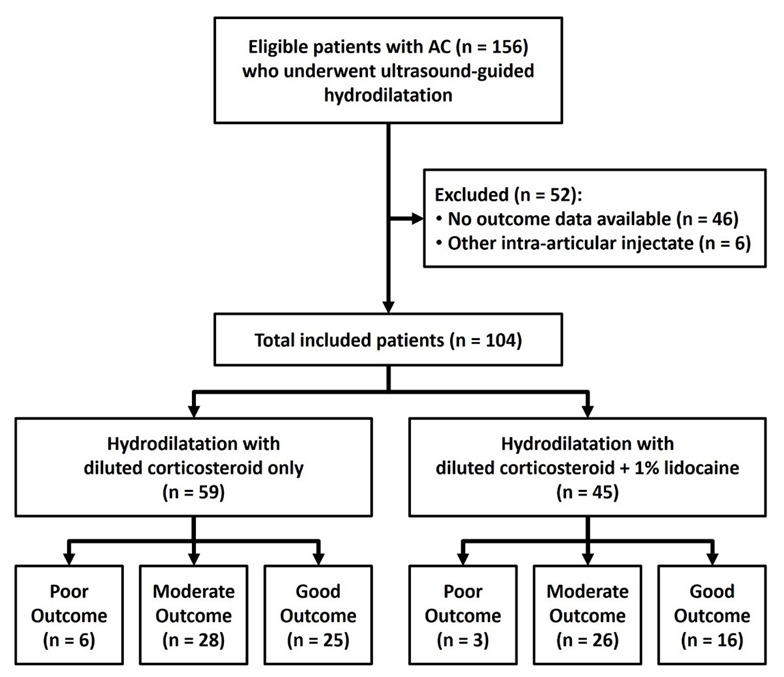
Retrospective study flow diagram of patients with adhesive capsulitis receiving ultrasound-guided hydrodilatation, with or without concomitant intra-articular lidocaine infusion. AC = adhesive capsulitis.

A total of 156 patients with AC fulfilled the inclusion criteria for this study (Figure 2). Among them, 46 patients were excluded due to incomplete documentation of overall treatment outcome, and 6 patients were excluded because they received an intra-articular injection with other types of injectate (4 patients with 15% dextrose and 2 patients with sodium hyaluronate). Ultimately, 104 patients (46 males and 58 females, mean age 55.7 ± 9.5 years, range 33 to 88 years) were included in the final analysis. Regarding the use of intra-articular lidocaine, 59 patients (30 males and 29 females, mean age 56.7 ± 10.3 years, range 33 to 88 years) underwent ultrasound-guided hydrodilatation with diluted corticosteroid only, and the other 45 patients (16 males and 29 females, mean age 54.4 ± 8.2 years, range 40 to 76 years) underwent ultrasound-guided hydrodilatation with mixed, diluted corticosteroid and 1% lidocaine. Levene’s test indicated equal variance in age distribution between the two groups (F = 1.351, *p* = 0.248). There were no significant group-wise differences in baseline demographics including age (t = 0.542, *p* = 0.589), gender (percentage of female patients, 49.2% vs. 64.4%; χ^2^ = 2.420 *p* = 0.120), and laterality of the affected shoulder (percentage of left shoulder, 57.6% vs. 55.6%; χ^2^ = 0.45, *p* = 0.833) (Table 1).

### 3.2. Treatment Outcome of Ultrasound-Guided Hydrodilatation with and without Lidocaine

**Figure 3:**
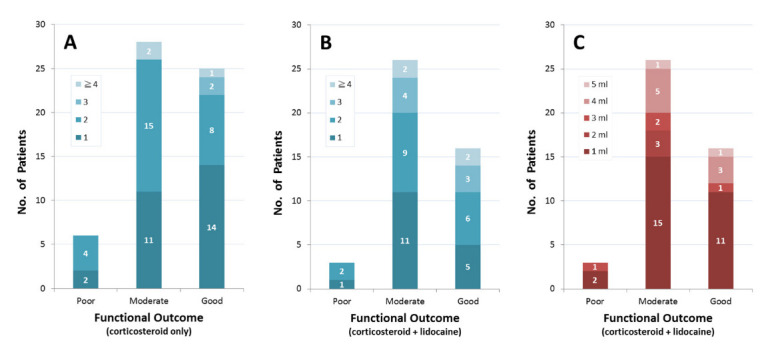
Distribution of patients by functional outcome and number of hydrodilatations in (**A**) patient group receiving hydrodilatation with diluted corticosteroid only and (**B**) patient group receiving hydrodilatation with diluted corticosteroid and 1% lidocaine. (**C**) Distribution of patients by functional outcome and received dose of intra-articular lidocaine in patient group receiving hydrodilatation with diluted corticosteroid and 1% lidocaine.

No significant group-wise differences in overall treatment outcome (U = 1273, *p* = 0.689) and number of hydrodilatations (U = 1126, *p* = 0.146) were found, with most patients showing moderate (28 patients vs. 26 patients, 47.4% vs. 57.8%) and good (25 patients vs. 16 patients, 42.4% vs. 35.5%) treatment outcomes after one to two courses of hydrodilatation, while only a few patients reported poor (6 patients vs. 3 patients, 10.2% vs. 6.7%) treatment outcome (Figure 3A,B). No significant correlation was found between the number of hydrodilatations and overall treatment outcome in all patients (r = −0.01, *p* = 0.916). Regarding the use of intra-articular lidocaine, most given patients were provided with 1 mL of 1% lidocaine (28 patients, 62%), followed by 4 mL (8 patients, 18%), 3 mL (4 patients, 9%), 2 mL (3 patients, 7%), and 5 mL (2 patients, 4%). Patients who received larger doses of lidocaine did not show greater treatment benefit (Figure 3C), and no significant correlation between the provided dose of intra-articular lidocaine and overall treatment outcome was found (r = −0.27, *p* = 0.858).

## 4. Discussion

The results of our retrospective study demonstrated that ultrasound-guided hydrodilatation is an effective treatment method for AC patients with increased short-term clinical benefits, regardless of concomitant intra-articular lidocaine use. The patient demographics of our study are consistent with previous studies in terms of gender predilection (more frequent in females), age distribution (more common in the middle-aged population), and tendency of involving the shoulder of the nondominant extremity [3]. The overall treatment outcome of our patients following ultrasound-guided hydrodilatation is also in agreement with a recent meta-analysis, suggesting that the combination of capsular distension and intra-articular corticosteroid injection can provide effective intervention for short-term symptom control [4]. To our knowledge, our study is the first to examine the necessity of intra-articular lidocaine infusion during the treatment process of ultrasound-guided hydrodilatation for patients with AC.

AC is known to cause local immune, inflammatory, or fibrotic changes at the glenohumeral joint due to primary idiopathic insults or occur secondarily as a complication following shoulder joint surgery [11,12]. Lidocaine has been widely used in the management of AC during hydrodilatation for both primary and secondary conditions in variable volume and concentration; meanwhile, intra-articular infusion of 0.5% lidocaine with volumes up to 19 mL has been previously reported [13,14,15,16,17]. Although intra-articular lidocaine can provide quick-acting analgesic support to help alleviate pain during treatment, there is cumulating evidence suggesting that lidocaine can induce chondrotoxicity with increased dosage and duration of exposure, and that it is more chondrotoxic than other local anesthetics [18,19,20]. Lidocaine was also found to potentiate the chondrotoxicity of corticosteroids toward human chondrocytes in an in vitro environment, although the underlying mechanism remains unknown [21]. On the other hand, previous in vivo and ex vivo studies by Ravnihar et al. demonstrated that single intra-articular lidocaine injections of the knee did not significantly affect chondrocyte viability, likely due to reduced synovial concentration following injectate dilution and diffusion, as well as protein binding [22,23]. However, it should be considered that the average joint volume of the knee is much greater than that of the glenohumeral joint, which is often significantly reduced during the disease course of AC to around 5–10 mL following capsular fibrosis, thickening, and contracture [24,25]; thus, it is more likely to retain or potentiate the chondrotoxic effects of lidocaine. Another study by Baumgarten et al. examined a cohort of AC patients treated via intra-articular injection with corticosteroid and local anesthetics including 1% lidocaine, and they reported no evidence of chondrolysis on the basis of radiographic, arthroscopic, and clinical findings during a nearly 5-year follow-up [26]. The authors did, nonetheless, emphasize the need for caution of use and continuous surveillance due to unaddressed issues, such as a possible delayed chondrolysis effect, and other chondrotoxic uncertainties related to drug volume and concentration. Taken together, there are currently insufficient clinical data or guidelines available to advise for or against the concomitant use of intra-articular lidocaine during hydrodilatation. Nevertheless, considering its transient analgesic benefit and potential chondrotoxic risks, intra-articular lidocaine should be used with discretion and adjusted according to clinical need and medical judgment.

On the other hand, the good overall clinical improvement of our patients without concomitant lidocaine use may be attributed to good operator experience and the technique of ultrasound-guided hydrodilatation performed. For the patients, we adopted a capsule-preserving strategy via an anterior rotator cuff interval approach that infuses a maximal injectate volume of 21 mL into the contracted glenohumeral joint capsule under ultrasound guidance. This technique is comparable to a previous method performed by Wang et al. [27], which allows for adequate capsular distension without the need of simultaneous pressure monitoring, thereby maximizing the duration of capsular expansion, as well as prolonging the anti-inflammatory effect of diluted corticosteroids and thus promoting successful treatment effects [28]. The use of the anterior rotator cuff interval approach is also known to deliver better treatment benefits in pain relief compared to the posterior approach [29], while good operator experience can promote successful hydrodilatation and enhance patient compliance.

This study has several limitations that need to be acknowledged. First, our study only included patients with idiopathic AC from a single center and was limited in case number. Further studies with larger patient cohorts, including those with secondary AC, are warranted to assess the generalizability of our results. Second, our study did not incorporate common assessment measures for AC, such as the shoulder pain and disability index (SPADI), shoulder disability index (SPI), and quantitative range-of-motion (ROM) measurements in the assessment of pretreatment baseline and post-treatment outcome, and we did not examine other relevant clinical information, including past medical history for diabetes, thyroid or autoimmune diseases, etc. This was limited by the retrospective nature of the present study and the available past outpatient medical information. Future study design should consider the routine use of a standardized medical record chart to keep relevant information available and updated. Nevertheless, the percentage of clinical improvement used in this study served as a simple and effective qualitative measure for assessing overall patient outcome, and it is reflective of real-world outpatient experience. Third, we did not differentiate between the patients and our treatment strategy according to the different stages of AC, which might have affected the study results. Future studies should consider the variable treatment effects of both lidocaine and corticosteroid at different stages of disease to facilitate better treatment planning. Finally, our study design did not incorporate the assessment of potential chondrotoxic effects of lidocaine among our patients, as no specific clinical information regarding lidocaine chondrotoxicity was available, and this is beyond the scope of this study. Nonetheless, currently, there is limited evidence to suggest that a single intra-articular injection of 1% lidocaine can cause clinically observable articular cartilage damage [6,26]. Other advanced assessment techniques such as non-invasive quantitative T2 mapping of the glenohumeral joint cartilage under magnetic resonance imaging (MRI) [29] may be considered in future studies.

In conclusion, the current study suggests that ultrasound-guided hydrodilatation without concurrent lidocaine infusion can deliver excellent treatment benefits for patients with AC, and it is supportive of a modified approach toward careful intra-articular lidocaine use during management of AC in the primary care setting. On the basis of the study results, we recommend that lidocaine should be provided on an as-needed basis during hydrodilatation instead of the conventional fixed-dose method. By combining a good hydrodilatation technique, appropriate analgesic strategy, and sufficient physiotherapy, we hope that this approach may promote good clinical treatment for patients with AC while obviating the risk of potential iatrogenic cartilage injury.

## Figures and Tables

**Table 1:** Comparison of baseline demographics and treatment outcome between the two groups.

	Total (n = 104)	Corticosteroid Only (n = 59, 56.7%)	Corticosteroid + Lidocaine (n = 45, 43.3%)	*p*-Value
Age (years) *	55.7 ± 9.5	56.7 ± 10.3	54.4 ± 8.2	0.589
Gender (Male/Female, Female %)	46/58 (55.8%)	30/29 (49.2%)	16/29 (64.4%)	0.120
Laterality (Left/Right, Left %)	59/45 (56.7%)	34/25 (57.6%)	25/20 (55.6%)	0.833
Treatment outcome (number, %)				
Poor	9 (8.7%)	6 (10.2%)	3 (6.7%)	0.689
Moderate	54 (51.9%)	28 (47.4%)	26 (57.8%)	
Good	41 (37.4%)	25 (42.4%)	16 (35.5%)	
No. of hydrodilatation (number, %)				
1	44 (42.3%)	27 (45.8%)	17 (37.8%)	0.146
2	44 (42.3%)	27 (45.8%)	17 (37.8%)	
3	9 (8.7%)	2 (3.4%)	7 (15.5%)	
≥4	7 (6.7%)	3 (5%)	4 (8.9%)	

* Levene’s test indicated equal variances (F = 1.351, *p* = 0.248).

## Data Availability

The data presented in this study are available on request from the corresponding author.

## References

[1] Neviaser A.S., Neviaser R.J. (2011). Adhesive capsulitis of the shoulder. J. Am. Acad. Orthop. Surg..

[2] Hand G.C., Athanasou N.A., Matthews T., Carr A.J. (2007). The pathology of frozen shoulder. J. Bone Joint. Surg. Br..

[3] Le H.V., Lee S.J., Nazarian A., Rodriguez E.K. (2017). Adhesive capsulitis of the shoulder: Review of pathophysiology and current clinical treatments. Shoulder Elbow.

[4] Challoumas D., Biddle M., McLean M., Millar N.L. (2020). Comparison of treatments for frozen shoulder: A systematic review and meta-analysis. JAMA Netw. Open..

[5] Piper S.L., Kramer J.D., Kim H.T., Feeley B.T. (2011). Effects of local anesthetics on articular cartilage. Am. J. Sports Med..

[6] Gulihar A., Robati S., Twaij H., Salih A., Taylor G.J. (2015). Articular cartilage and local anaesthetic: A systematic review of the current literature. J. Orthop..

[7] Hynes J.P., Kavanagh E.C. (2022). Complications in image-guided musculoskeletal injections. Skeletal Radiol..

[8] Dragoo J.L., Braun H.J., Kim H.J., Phan H.D., Golish S.R. (2012). The in vitro chondrotoxicity of single-dose local anesthetics. Am. J. Sports Med..

[9] Rymaruk S., Peach C. (2017). Indications for hydrodilatation for frozen shoulder. EFORT Open Rev..

[10] Catapano M., Mittal N., Adamich J., Kumbhare D., Sangha H. (2018). Hydrodilatation with corticosteroid for the treatment of adhesive capsulitis: A systematic review. PM R.

[11] Fama G., Tagliapietra J., Belluzzi E., Pozzuoli A., Biz C., Ruggieri P. (2021). Mid-Term Outcomes after Arthroscopic “Tear Completion Repair” of Partial Thickness Rotator Cuff Tears. Medicina.

[12] Ryan V., Brown H., Minns Lowe C.J., Lewis J.S. (2016). The pathophysiology associated with primary (idiopathic) frozen shoulder: A systematic review. BMC Musculoskelet. Disord..

[13] Shah N., Lewis M. (2007). Shoulder adhesive capsulitis: Systematic review of randomised trials using multiple corticosteroid injections. Br. J. Gen. Pract..

[14] Wu W.T., Chang K.V., Han D.S., Chang C.H., Yang F.S., Lin C.P. (2017). Effectiveness of glenohumeral joint dilatation for treatment of frozen shoulder: A systematic review and meta-analysis of randomized controlled trials. Sci. Rep..

[15] Lin M.T., Hsiao M.Y., Tu Y.K., Wang T.G. (2018). Comparative efficacy of intra-articular steroid injection and distension in patients with frozen shoulder: A systematic review and network meta-analysis. Arch. Phys. Med. Rehabil..

[16] Rex S.S., Kottam L., McDaid C., Brealey S., Dias J., Hewitt C.E., Keding A., Lamb S.E., Wright K., Rangan A. (2021). Effectiveness of interventions for the management of primary frozen shoulder: A systematic review of randomized trials. Bone Jt. Open..

[17] Makki D., Al-Yaseen M., Almari F., Monga P., Funk L., Basu S., Walton M. (2021). Shoulder hydrodilatation for primary, post-traumatic and post-operative adhesive capsulitis. Shoulder Elbow.

[18] Jayaram P., Kennedy D.J., Yeh P.L., Dragoo J. (2019). Chondrotoxic effects of local anesthetics on human knee articular cartilage: A systematic review. PM R.

[19] Kreuz P.C., Steinwachs M., Angele P. (2018). Single-dose local anesthetics exhibit a type-, dose-, and time-dependent chondrotoxic effect on chondrocytes and cartilage: A systematic review of the current literature. Knee Surg. Sports Traumatol. Arthrosc..

[20] Karpie J.C., Chu C.R. (2007). Lidocaine exhibits dose- and time-dependent cytotoxic effects on bovine articular chondrocytes in vitro. Am. J. Sports Med..

[21] Braun H.J., Wilcox-Fogel N., Kim H.J., Pouliot M.A., Harris A.H., Dragoo J.L. (2012). The effect of local anesthetic and corticosteroid combinations on chondrocyte viability. Knee Surg. Sports Traumatol. Arthrosc..

[22] Ravnihar K., Marš T., Pirkmajer S., Alibegović A., Koželj G., Stožer A., Drobnič M. (2021). The influence of a single intra-articular lidocaine injection on the viability of articular cartilage in the knee. Cartilage.

[23] Ravnihar K., Barlič A., Drobnič M. (2014). Effect of intra-articular local anesthesia on articular cartilage in the knee. Arthroscopy.

[24] Matziolis G., Roehner E., Windisch C., Wagner A. (2015). The volume of the human knee joint. Arch. Orthop. Trauma. Surg..

[25] Nagy M.T., Macfarlane R.J., Khan Y., Waseem M. (2013). The frozen shoulder: Myths and realities. Open Orthop. J..

[26] Baumgarten K.M., Helsper E. (2016). Does chondrolysis occur after corticosteroid-analgesic injections? An analysis of patients treated for adhesive capsulitis of the shoulder. J. Shoulder Elbow Surg..

[27] Wang J.C., Tsai P.Y., Hsu P.C., Huang J.R., Wang K.A., Chou C.L., Chang K.V. (2021). Ultrasound-Guided Hydrodilatation with triamcinolone acetonide for adhesive capsulitis: A randomized controlled trial comparing the posterior glenohumeral recess and the rotator cuff interval approaches. Front. Pharmacol..

[28] Cho J.H. (2021). Updates on the treatment of adhesive capsulitis with hydraulic distension. Yeungnam Univ. J. Med..

[29] Lockard C.A., Nolte P.C., Gawronski K., Elrick B.P., Goldenberg B.T., Horan M.P., Dornan G.J., Ho C.P., Millett P.J. (2021). Quantitative T2 mapping of the glenohumeral joint cartilage in asymptomatic shoulders and shoulders with increasing severity of rotator cuff pathology. Eur. J. Radiol. Open.

